# Roughness measurement of leaf surface based on shape from focus

**DOI:** 10.1186/s13007-021-00773-y

**Published:** 2021-07-09

**Authors:** Zeqing Zhang, Fei Liu, Zhenjiang Zhou, Yong He, Hui Fang

**Affiliations:** 1 College of Biosystems Engineering and Food Science, Zhejiang University, Hangzhou, China; 2 Huzhou Insititute of Zhejiang University, Huzhou, China

**Keywords:** Shape from focus, 3d-reconstruction, Surface roughness

## Abstract

**Background:**

Surface roughness has a significant effect on leaf wettability. Consequently, it influences the efficiency and effectiveness of pesticide application. Therefore, roughness measurement of leaf surface offers support to the relevant research efforts. To characterize surface roughness, the prevailing methods have drawn support from large equipment that often come with high costs and poor portability, which is not suitable for field measurement. Additionally, such equipment may even suffer from inherent drawbacks like the absence of relationship between pixel intensity and corresponding height for scanning electron microscope (SEM).

**Results:**

An imaging system with variable object distance was created to capture images of plant leaves, and a method based on shape from focus (SFF) was proposed. The given space-variantly blurred images were processed with the proposed algorithm to obtain the surface roughness of plant leaves. The algorithm improves the current SFF method through image alignment, focus distortion correction, and the introduction of NaN values that allows it to be applied for precise 3d-reconstruction and small-scale surface roughness measurement.

**Conclusion:**

Compared with methods that rely on optical three-dimensional interference microscope, the method proposed in this paper preserves the overall topography of leaf surface, and achieves superior cost performance at the same time. It is clear from experiments on standard gauge blocks that the RMSE of step was approximately 4.44 µm. Furthermore, according to the Friedman/Nemenyi test, the focus measure operator SML was expected to demonstrate the best performance.

## Background

In light of concerns for the environment, resource and costs, the improvement of pesticide efficiency is a classical issue in agricultural engineering. Despite the variance in the definition of leaf wettability [1], in this paper, the term refers to the manifestation of submicron physicochemical interactions between leaf surface and solution droplet [2], i.e. the affinity of leaves to water or medicine. There exists a large number of studies on leaf wettability that aim to enhance the adhesion of droplets on the surface of leaves, thereby averting the off-target deposits (e.g. rebound, roll, slide) that result from the adhesive characteristics of leaf surface. Leaf wettability may differ significantly among different species, varieties, and may even vary during the different stages of a life cycle [3]. Plant leaves, when observed at a high resolution, are rarely absolutely flat. Previous studies found it is the chemical composite and microstructure of epicuticular wax formations on leaves that determine leaf wettability. Increases in the surface roughness of a hydrophobic surface normally lead to increases in the hydrophobic properties [4]. Contact angle is the angle where a liquid–vapor interface meets a solid surface (plant leaf in this case). Though contact angle is a common metric of leaf wettability, its contribution to intensive studies on wettability is very limited. When quantifying the separate factors of leaf wettability, instead of contact angle, surface roughness is used as a viable index, as wettability is a function of roughness.

In the manufacturing industry, surface roughness characterization is standardized, whereas, unfortunately, a mature method for surface roughness characterization of leaves is lacking [5]. Pioneers of the field attempted to account for contact angle as a parameter in roughness coefficient [6, 7] by contrasting the contact angle of rough surfaces with that of smooth surfaces, where the roughness coefficient is merely a relative and indirect assessment of roughness. Scanning electron microscope (SEM) provides high-resolution micrograph of leaf surface, and with the help of computer vision techniques (e.g. fractal dimensional analysis [1, 8, 9], Fourier descriptors [10, 11], visual classification [12, 13]), it is one of the viable options for obtaining leaf surface roughness. However, before inspection using SEM, the preprocessing of samples is cumbersome. Besides, the computer vision techniques based on two-dimensional SEM micrograph require significant correlation between surface texture and roughness. Atomic force microscope (AFM) can be used for highly accurate probing of the surface profile of leaves through a stylus [4, 14]. The adoption of AFM, like most of stylus-enabled methods of characterizing surface roughness in the manufacturing industry, may damage the surface, despite the creation of ameliorated non-contact AFM that aims to alleviate the problem at the cost of accuracy. Besides, AFM can only gain access to one-dimensional profile within an action cycle, and its *z*-range for leaves of many plant species is unacceptably narrow. Optical profiler, a profiler based on optical principles such as phase shifting interferometry, was used to render the surface of plant leaves [5, 15]. Using interferometry, speckles formed by white light interferer compose optical sections, and then the leaf surface can be reconstructed from these sections. This procedure is often automated and faster, but it may omit the most valuable information concerning the spatial organization of a surface [11].

The prevailing methods for characterizing leaf surface roughness, dependent on expensive instruments, have to deal with a number of shortcomings. To address the issue, this study proposes a new approach to measure the leaf surface roughness based on shape from focus (SFF). As a textured rough surface moves with respect to a fixed imaging system, variation of image sharpness is observed, enabling the recovery of the shape from the textured image. In this study, in order to reconstruct the surface topography where surface roughness is recovered, micrograph sequences of leaves captured by high-resolution digital camera with diverse focusing deviation are processed by the SFF algorithm. Cost of the method proposed in this paper is notably lower compared to the previous methods. Moreover, it utilizes 3-demensional information and features noncontact measurement process. To evaluate performance of the method, results of the optical profiler are specified hereunder as reference.

## Materials and methods

### Materials

Customized steel gauge blocks that are precisely 1000 µm to 1100 µm ± 0.2 µm thick at intervals of 10 µm function as the reference standard. The pairwise combination of gauge blocks yielded 10 measurement results at different step height: from 10 to 100 µm by 10 µm. Leaves of lhe hybrid indica rice (*Oryza sativa* L.), grown from seed in an incubator, were sampled at seedling stage. The Rape (*Brassica chinensis* L.) was Zheda 618, which was bred by Zhejiang University. The cotton (*Gossypium hirsutum* L.) was upland TM-1. The tobacco (*Nicotiana tabacum* L.) was benthamiana.

**Fig. 1:**
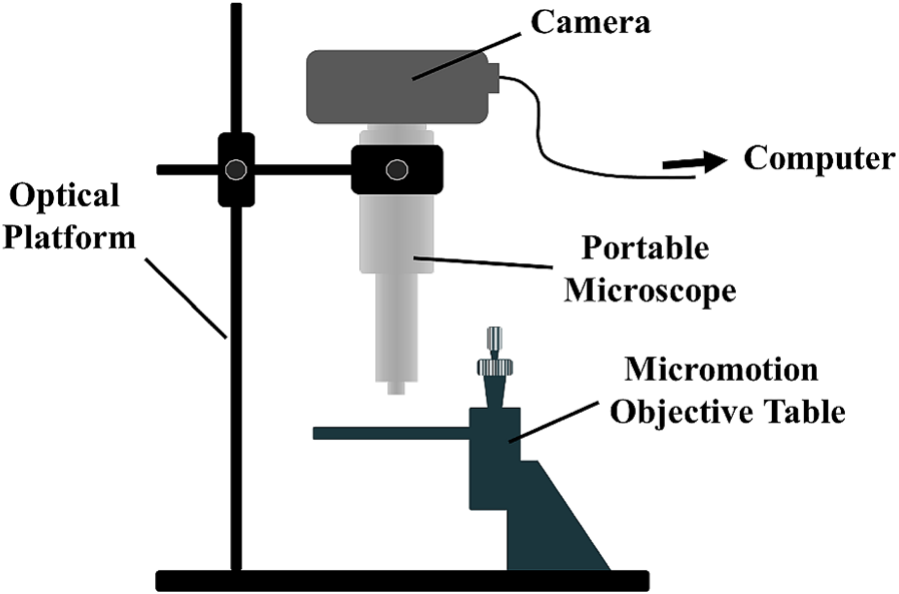
The shape from focus imaging system

A SFF imaging system (Fig. 1) was built in Agricultural Information Technology Institute of Zhejiang University (Hangzhou, PRC) to capture focused and defocused images. The system comprised an optical experimental platform (Fenghua Technology Co., Ltd, Shenzhen, PRC), an industrial RGB camera (Tuoriweiye Technology Co., Ltd, Shenzhen, PRC), a portable microscope (Meijing Electronics Co., Ltd, Shanghai, PRC), and a micromotion objective table (Hengyang Electronic Technology Co., Ltd, Guangzhou, PRC). The resolution of the RGB camera is 3072 pixels $\times$ 2048 pixels. The portable microscope, functioning as a camera lens, is capable of magnification of $\times 10$. The micromotion objective table, installed on the optical experimental platform, shifts leaf vertically in the motion range of 12.5 mm and accuracy of 0.5 µm. Manual adjustment to ratchet knob lifts the table in precision, thus creating defocus. The camera was mounted on the portable microscope, upside down towards the table, and the images were transmitted to computer via Ethernet.

Wyko NT9100 (Veeco, NY, US) optical profiler was utilized to measure leaf roughness as a comparison. This instrument, suitable for measuring 3d surface topography, has a vertical scanning range of 0.1nm–10 mm. The measurement was made in College of Optical Science and Engineering, Zhejiang University (Hangzhou, PRC), where the optical profiler was located. Upon measurement, the defocused images were immediately captured, and the leaves were moved to the optical profiler for observation. Thanks to the supporting software, the three-dimensional structure was reconstructed and the surface roughness was obtained with ease.

### Shape from focus

The notion of shape from focus, also known as depth from focus (DFF), is hardly new. It involves a classic problem in computer vision. SFF is widely studied as a passive method of estimating depth or shape from monocular focal cues [16–19].

**Fig. 2:**
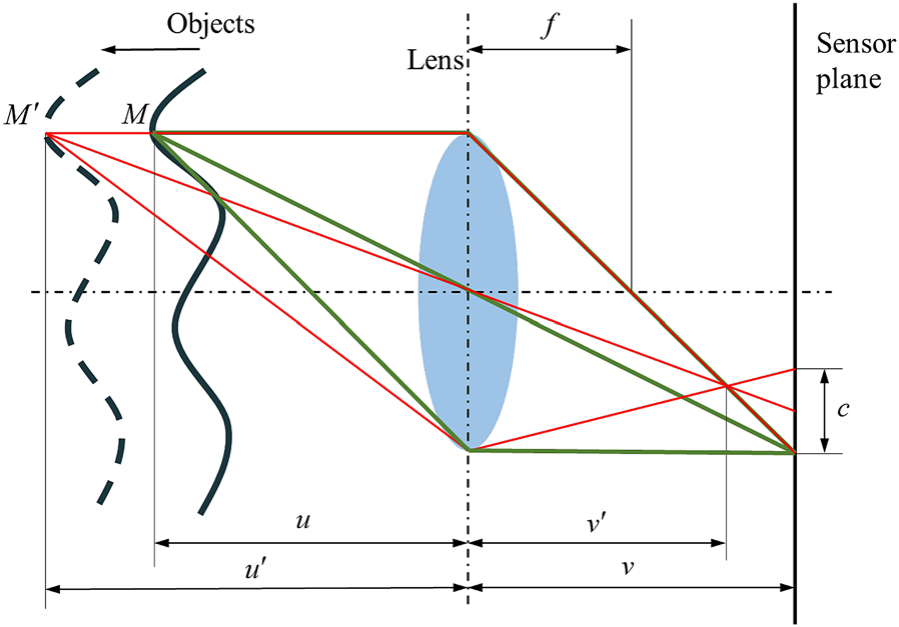
Convex lens model with objects motion along with *z*-axis

With a fixed setting of focus length, observations along the *z*-axis are different in blurriness, which is one of the most obvious cues for human observers to understand depth in a two-dimensional image. As to the microscopic level, when projecting to the sensor plane (Fig. 2), the radiation of each point on the object spread onto a fuzzy circle, which is usually expressed by point spread functions (PSFs). In particular, if the focus locates on the sensor plane, the diameter of the fuzzy circle is infinitely close to zero. In this case, since the sensor plane and the focal plane are coincident, the projection of point *M* is an ideal point. For a constant focal length *f* and a fixed location of lens and sensor plane, when the objects move away from the lens, the focus of the point $M^{\prime}$ moves towards the lens, and a fuzzy circle with the diameter of *c* is synchronously detected on the sensor plane.

Methods known as depth from defocus (DFD) or shape from defocus (SFD) aim to compute the distance between the defocused object points and the sensor plane by estimating the diameter of fuzzy circles. These methods are speedy but inaccurate. Furthermore, when adopting them, intrinsic and extrinsic parameters must also be determined in advance. According to SFF theories, a focused image surface (FIS) is defined as the surface formed by a set of points at which the object points are focused by the lens [20]. FIS is determined on the basis of the sharpness at each pixel in a sequence of images with constantly varying focus levels, and a focus measure function or operator is applied to compute the sharpness. Conventionally, FIS comprises pixels where the corresponding frame gives the maximal sharpness among the images, but it is also true that certain applications feature optimized techniques (e.g. quadratic interpolation, Gaussian interpolation) for smooth surfaces. For example, quadratic interpolation method determines a quadratic function by 3 maximal focus measures, and the final maximal sharpness should be ideally located at the axis of symmetry. When FIS is available, all corresponding points on the object surface can be retrieved.

### Image registration

Image registration focuses on aligning two or more images of the same scene through geometrical warping and overlapping. Research schemes of most SFF studies ignore the differences in view-of-field during the adjustment of focus level, as image registration comes with considerable costs. However, this study proposes the correction of the pixel offset, while taking into account the trade-off between the accuracy requirement and the computational complexity in roughness measurement of leaf surface. An illustration of the image registration process is given in the following paragraphs.

First, the relevant features are detected using Speed-Up Robust Feature (SURF) [21]. Owing to its rapidity and robustness, SURF has extensive applications in image registration. The strategies in relation to Gaussian pyramid and Harr wavelet make the SURF descriptor scale- and rotation-invariant. In vast image registration tasks, SURF outperformed other prevailing methods. Thus, this paper proposes the use of SURF as the feature detector in this scheme. For each image, SURF algorithm yields dozens of features expressed by 128-deminsional vectors.

Second, image features are matched based on the correspondence between the features within pairs of images. The so-called correspondence is a function, which characterizes the spatial relationship, of two features. As a frequently used distance, Euclidean distance was adopted in this scheme. By calculating the distance of every pair of features between neighbor frames, the brute-force matches can be obtained. The amount of matches indicates the clarity of the images to some extent. Points on images with few matches are largely defocused, which is not appropriate for SFF. Therefore, for the sake of arithmetical simplification in subsequent procedures, the images with insufficient matches were removed from the dataset, and the threshold $\eta$ was set according to the texture richness of leaf surface.

Then, homography matrices are estimated from the matches. Theoretically, the distortion of images resulting from the adjustment of focal level is nothing but scaling, yet in practice, it is clear that miscellaneous errors may incur other forms of distortion. The homography matrix denotes the mapping relation between two image coordinates, which is a 3 $\times$ 3 matrix, and is independent of scalar multiplication. Thus, 8 parameters should be estimated for each homography matrix, i.e. at least 4 matches are needed before an image can be transformed. In addition, Random Sample Consensus (RANSAC) works as a filter of matches, considering the high sensitivity of homography to noise.

At last, the homography matrices are applied to image transformation. In this stage, all of the remaining images (meeting the limit of the aforementioned threshold $\eta$) are transformed into the same image coordinate (e.g. that of the first image). Linear interpolation is used to deal with non-integer pixel indices that occurred due to the spatial discreteness of digital images. After the transformation, the pixel offsets among the image frames are eliminated so that an overlapping region emerges. For each image frame, the edge outside the overlapping region should be trimmed off, thus ensuring that there is no absent pixel.

### Focus measure

Focus measure plays a dominant role in SFF since it produces information that are fundamental for FIS. A great number of focus measure operators have been proposed in previous studies. Pertuz et al. [17] offered a summary of these focus measure operators by grouping them into 6 families. Though it is difficult to determine which operator family performs best under any given imaging conditions, the Laplacian family demonstrates the best performance overall. Some operators working in frequency domain, widely used in autofocus, also showed sound performance results; nevertheless, the time-consuming Fourier transformation offers little support to solving SFF problems since focus measure is determined in local windows.

In this paper, performances of four spatial focus measure operators were compared.

#### Energy of Laplacian (EL)

Energy of Laplacian considers the second derivative of images. It is calculated by summing the pixel intensity of an image convolved with a Laplacian mask. This step is carried out within a local window for denoising purpose, which is expressed by the following formula:

1 $$\text {EL}(u,v)= \sum _{(x,y)\in \Omega (u,v)}\mathcal {L}(x,y),$$

2 $$\mathcal {L}(x,y)= \frac{\partial ^{2}f(x,y)}{\partial x^{2}}+\frac{\partial ^{2}f(x,y)}{\partial y^{2}}.$$

wherein, $\mathcal {L}(\cdot )$ denotes Laplacian transformation; $f(\cdot )$ denotes the pixel intensity of image; and $\Omega (\cdot )$ denotes the adjacent region (same below).

#### Sum-modified Laplacian (SML)

Laplacian operator calculates the second partial derivatives of images with respect to *x* and *y*, which can be either positive or negative. Therefore, Energy of Laplacian may show a small response at pixels where the two partial derivatives at orthogonal directions cancel out. Different from Energy of Laplacian, Sum-modified Laplacian sums the energy of a window for an image convolving with a modified Laplacian (ML) operator, which is defined as the sum of absolute value of second partial derivatives:

3 $$\text {SML}(u,v)= \sum _{(x,y)\in \Omega (u,v)}\mathcal {L}_{m}(x,y),$$

4 $$\mathcal {L}_{m}(x,y)= \left| \frac{\partial ^{2}f(x,y)}{\partial x^{2}}\right| +\left| \frac{\partial ^{2}f(x,y)}{\partial y^{2}}\right| .$$

wherein, $\mathcal {L}_{m}(\cdot )$ denotes modified Laplacian transformation.

#### Tenenbaum gradient (TG)

Tenenbaum [22] proposed a focus measure operator based on Sobel operator, which is named Tenenbaum gradient. Sobel operator, known as an edge detector, has two forms which produce the first derivative of images with respect to *x* and *y*, respectively. By summing the length of gradient within a window, Tenenbaum gradient is obtained by the following formula:

5 $$\text {TG}(u,v)= \sum _{(x,y)\in \Omega (u,v)}G(x,y),$$

6 $$G(x,y)= \sqrt{G_{x}^{2}(x,y)+G_{y}^{2}(x,y)}.$$

#### Gray level variance (GLV)

Gray level variance is a statistic operator based on the assumption that points at FIS come with the maximal gray level variance. Naturally, GLV is also calculated in local windows:

7 $$\text {GLV}(u,v)=\sum _{(x,y)\in \Omega (u,v)}(f(x,y)-\mu )^2.$$

wherein, $\mu$ denotes the mean pixel intensity of the window.

### FIS searching

The principal and most straightforward way to determine FIS is to search for the maxima of focus measure at each pixel [23]. Though implementation of such an approach is both fast and simple, the resolution of FIS depends on the movement interval of object with respect to the image detector, i.e. the reconstructed surface looks discrete. A great many of SFF studies inclines to interpolate the points nearby the maximum. In such studies, effective interpolation techniques including quadratic [24] and Gaussian [25] interpolation have been extensively adopted. Interpolation methods aim at smoothing FIS, yet, like the former approach, a maxima caused by noise may lead to its distortion. Besides, though closed form solutions can be provided for the specific situations, interpolation methods are often time-consuming. Some researchers optimized the FIS using tensor voting [26, 27] but the algorithm iterates every token from depth cloud and computes the eigenvalues, which is not favorable to addressing the current problem.

This paper proposed a simple but efficient and robust method for FIS search using weighted average of stereo blurred focus measure.

The values of focus measure for each pixel were obtained by applying the aforementioned focus measure operators according to the specified procedures. Considering the potentially abnormal values (due to noise), the original values of focus measure were blurred with a stereo mean filter.

The blurred focus measure is defined as:

8 $$\text {BFM}_k(i,j)=\frac{1}{N}\sum _{(x,y,z)\in \Omega '(i,j,k)}\text {FM}_z(x,y).$$

wherein, *N* denotes the number of pixels in the window; *k* denotes the image frame of the focus measure; $\text {FM}_z$ (x,y) denotes the focus measure at pixel (*x*, *y*) for image frame *z*; and $\Omega^{\prime}(\cdot )$ denotes the adjacent region of the pixel over frames. Here, $\Omega^{\prime}(\cdot )$ is a $3\times 3\times 3$ window.

In order to achieve fast and precise FIS search, information of the focus measure should be fully utilized without increasing the difficulty of computation. With respect to pixels in different image frames at the same pixel coordinate, values of focus measure were sorted from largest to smallest. Then, FIS is calculated by the following formula:

9 $$\text {FIS}(i,j)= {\left\{ \begin{array}{ll} \text {NaN}, & \sigma _\varphi > \lambda m\\ \frac{\delta \sum _{k\in \varphi _m}k\text {BFM}_k(i,j)}{\sum _{k\in \varphi _m}\text {BFM}_k(i,j)}, & \sigma _\varphi \le \lambda m \end{array}\right. },$$

wherein, $\varphi _m$ denotes the first *m* frame numbers of sorted focus measure values; $\delta$ denotes the interval distance between neibour frames; and $\sigma _\varphi$ denotes the standard deviation of $\varphi _m$. The coefficient $\lambda$ is a manual setting parameter. The experimental value of $\lambda$ is 0.5. $\text {NaN}$ (Not a Number) represents an invalid value.

**Fig. 3:**
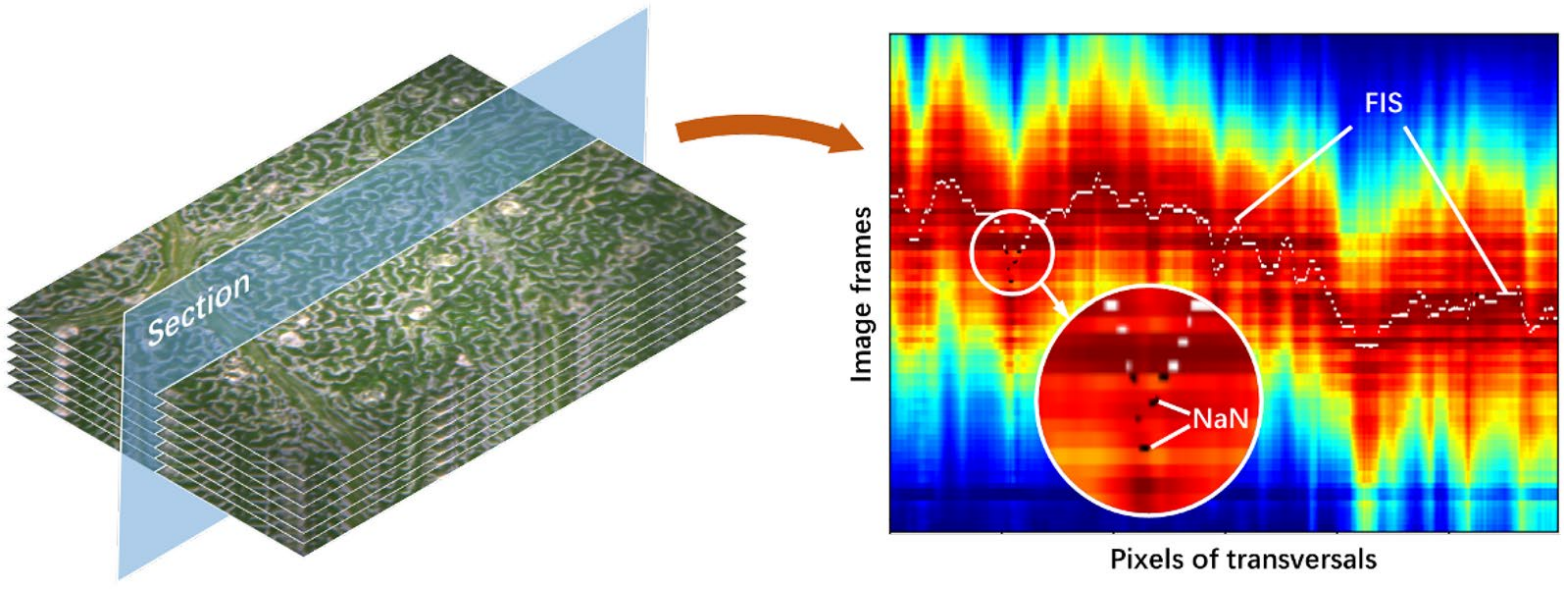
FIS searching on the section of cotton leaf image frames. Focus measure of the transversal are shown using pseudo color map, which increases as tone turns from cool to warm. Flecked with black points marked as NaN, the curve formed by white points is the FIS to search

Formula (9) indicates that FIS is determined by the weighted average of $\varphi _m$, where the blurred focus measure functions as the weight. The method proposed in this paper considers the score of image frame where FIS can possibly be found, and yields the comprehensively predicted location. A large $\sigma _\varphi$ indicates the unreliability of focus measure. Therefore, fixing the invalid values for FIS on these pixels is an approach to prevent them from getting involved in the subsequent computation. Figure 3 shows the performance of the proposed FIS search method on cotton leaf surface. It is evident that this method is less sensitive to maximum compared with interpolation methods.

**Fig. 4:**
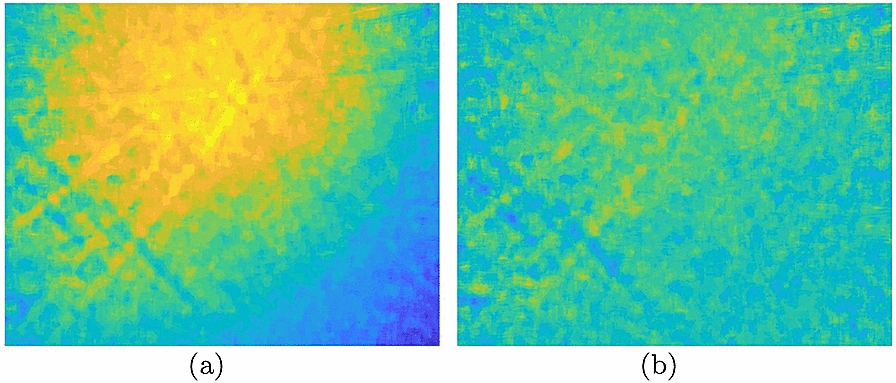
Pseudo-color map of FIS comparison before and after correction by a drift field. Pixel height increases as tone turns from cool to warm. **a** Distorted FIS. **b** Undistorted FIS

In most cases of application, the SFF procedures will end upon finding FIS, and the subsequent measurement of surface feature parameters is taken for granted. In practice, an abnormal bulge emerges from the central region of the reconstructed surface. Drift of FIS is potentially ubiquitous in SFF due to defects in relation to the fabrication of camera, the assembly accuracy of microscope and the sensitivity discrepancies of sensing units. For example, if the lens is not in ideal shape, the lights, which should have intersected on the sensor plane, will intersect forward or backward. It will therefore result in a SFF offset. Although this effect is often negligent, it becomes significant in microscopic applications. Essentially, the distortion can be regarded as the manifestation of barrel distortion in depth. Universality of barrel distortion in short focus lens accounts for the significance of FIS distortion in microscopic applications. To eliminate such distortion, the difference of the primary FIS and a drift field substitute the primary FIS, in which the drift field denotes the strongly blurred FIS of a flat surface. This is not a cumbersome process of duplication, as the measure of the drift field, i.e., system calibration, is completed once for all upon establishment of the system. Figure 4 demonstrates the effective correction of a flat surface with scratches of aluminum alloy. It is clear that an apparently abnormal uplift of FIS is restrained in the undistorted image.

## Results and discussion

### Comparison of surface rendering

**Fig. 5:**
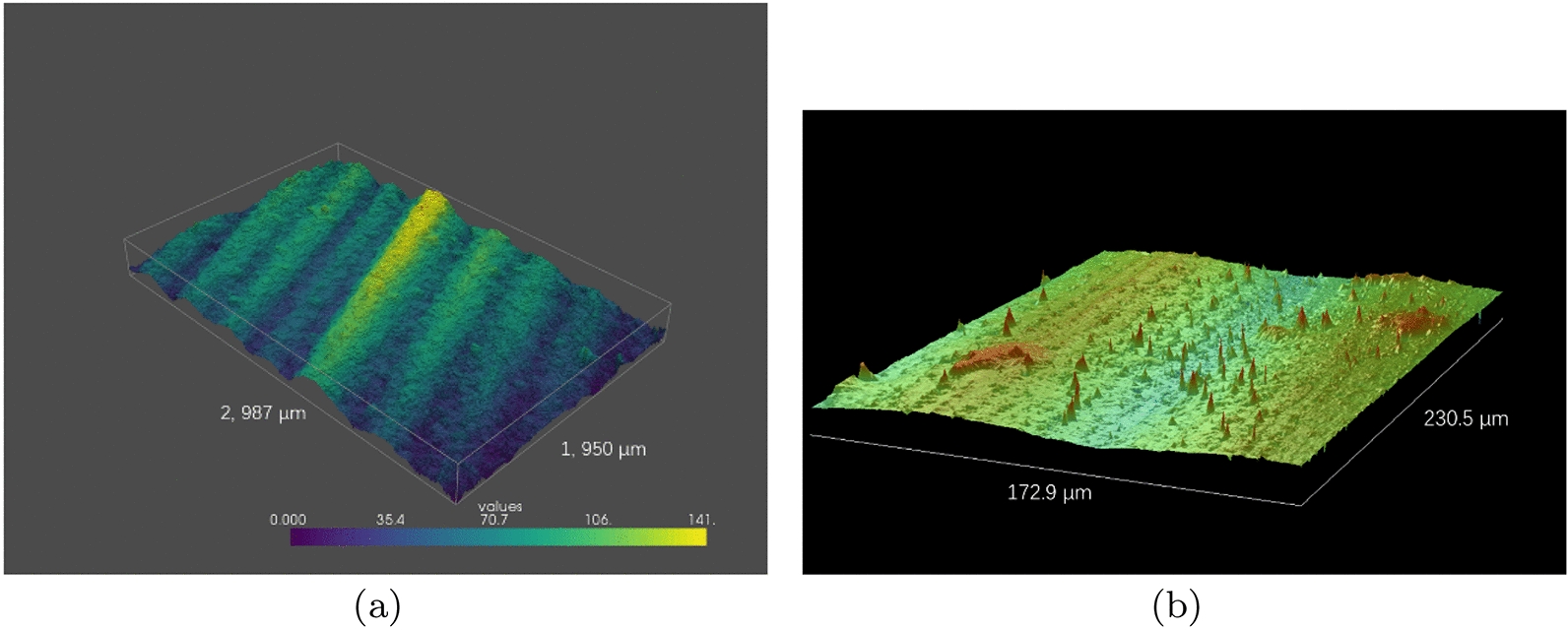
Computer rendering of rice leaves. **a** SFF rendering. **b** Optical profiler rendering

Plant leaves of various species were observed in both optical profiler and the SFF system. Figure 5 compares the surface renderings of these two methods. In this comparison, the height of surface gets larger as the color shifts from cool tone to warm tone, and the SML operator was chosen for focus measure in SFF renderings. It should be noted that it was very challenging to present the identical region of interest in the two methods due to differences in field of view. Thus, a qualitative evaluation had be conducted according to the comparison of overall performances.

Plant leaves normally present fractal structures. As is shown in Fig. 5a, b, the surface of rice leaf comprises complex microstructures including parallel wavy veins, hairy trichomes, quasi-1D arranged micropapillae and patchy protuberances. An inherent drawback of SFF lies in its mediocre performance for details and abrupt changes due to local windows in focus measure. In the SFF rendering, comparatively speaking, theses details are suppressed; certain detailed information are preserved nonetheless. SFF rendering demonstrates a distinctly higher altitude at areas where the trichomes and protuberances were expected. Relatively speaking, the veins are well defined in SFF reconstruction since larger scale of scenes are less sensitive to the local windows.

It is evident that the field of view of optical profiler is much smaller than that of the SFF system. The optical profiler renders no more than one vein per observation, while the SFF system offers a rendering of several veins thanks to its advantage of a wider field of view. Since the wavy veins on rice leaves are considerably different in size, the accuracy of roughness measurement for optical profiler is largely dependent on the selection of single vein. By contrast, such dependence abates in the SFF system.

**Fig. 6:**
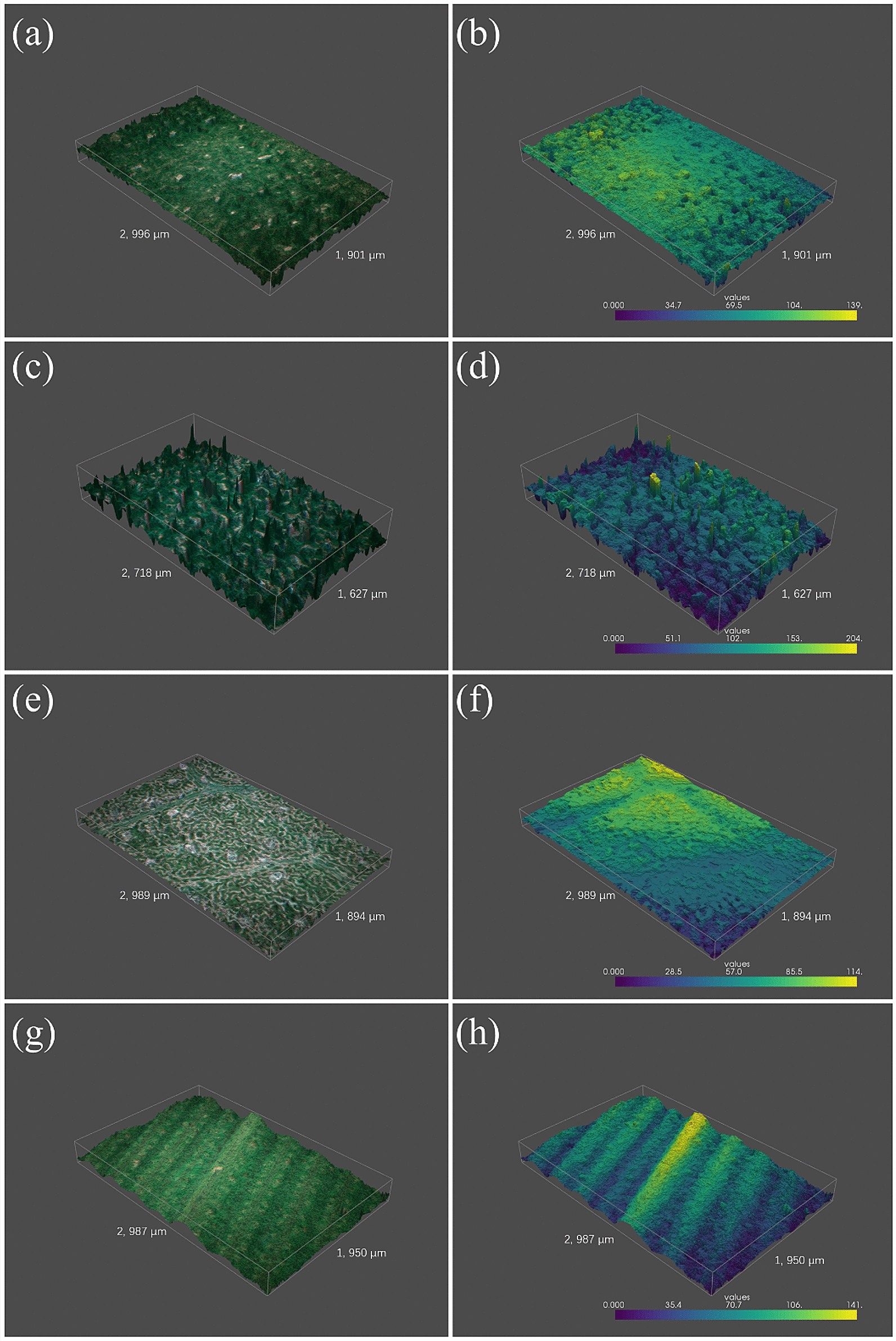
Computer renderings of leaf surface of different species (rape, tobacco, cotton and rice from top to bottom). The first column is Computer renderings with texture. The second column is Computer renderings with depth in pseudo color map

Figure 6 shows the computer renderings of rape, tobacco, cotton and rice leaf surface. Dicotyledons are characterized by reticular veins. Leaf surface trends to be flat where the veins are sparse, a fact that has been well demonstrated by the renderings.

Table 1 shows the areal roughness of plant leaf surface displayed in Fig. 6. If *A* is the area where the roughness is measured, then the areal surface roughness based on arithmetical mean height is calculated by the following formula:

10 $$S_a=\frac{1}{A}\int _{(x,y)\in A}\left| \text {FIS}(x,y)\right| \,\text {d}x\,\text {d}y.$$

**Table 1:** Areal surface roughness of the plant leaves

Plant leaf	*S*_*a*_/µm
Rape	7.94
Tobacco	24.43
Cotton	16.75
Rice	18.86

### Quantitative analysis on gauge blocks

**Fig. 7:**
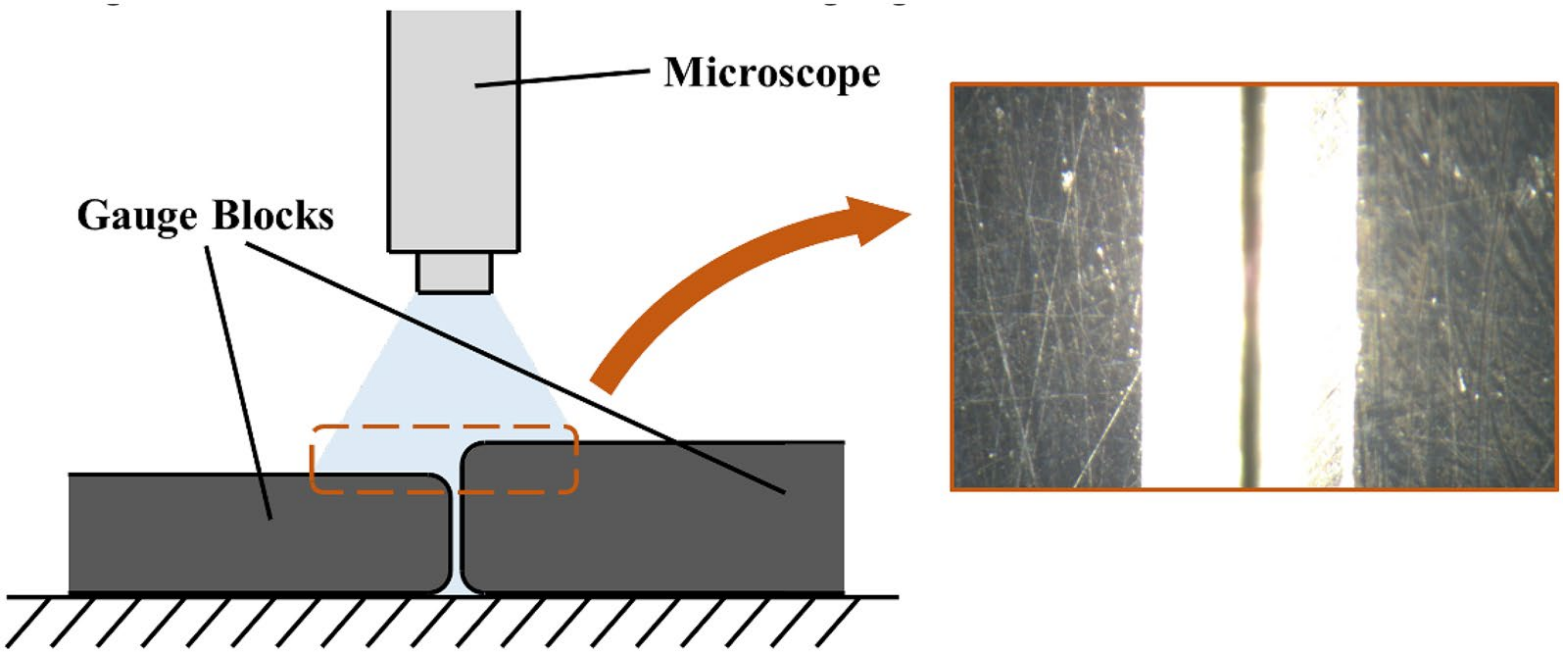
Diagrammatic sketch of FIS measure on gauge blocks

The measurement of identical micro-surface profile on different instruments is quite a challenge. In order to quantify the performance of the SFF system for each FIS method, experiments were conducted via pairwise combination of the gauge blocks along their edges, creating a step at the shared border. Figure 7 shows the diagram of experimental measure where the microscope aimed at the seam of the blocks. The pairwise combination of gauge blocks yields 10 measurements at different step height: 10–100 µm at intervals of 10 µm. For each measurement, approximately 10,000 samples were selected from the FIS at the two step faces, respectively, and the height of the step was calculated. The number of samples depends on the alignment and NaN values since the sampling was conducted in columns. Root Mean Square Error (RMSE) and Pearson correlation were computed using ground truth and reconstructed depth map. The smaller the RMSE and the more significant the correlation, the better the performance. If *f*(*m*, *n*) and *h*(*m*, *n*) are the computed value and the actual value of the step height for the *n*-th sample of the *m*-th measurement, then the RMSE and the Pearson correlation *r* are expressed by the following formula:

11 $$\text {RMSE}= \sqrt{\frac{1}{MN}\sum _{m=1}^{M}\sum _{n=1}^{N}\left( f\left( m,n\right) -h\left( m,n\right) \right) ^2},$$

12 $$r= \frac{\sum _{m=1}^{M}\sum _{n=1}^{N}\left( f\left( m,n\right) -\bar{f}\right) \left( h\left( m,n\right) -\bar{h}\right) }{\sqrt{\left( \sum _{m=1}^{M}\sum _{n=1}^{N}\left( f\left( m,n\right) -\bar{f}\right) \right) \left( \sum _{m=1}^{M}\sum _{n=1}^{N}\left( h\left( m,n\right) -\bar{h}\right) \right) }}.$$

**Fig. 8:**
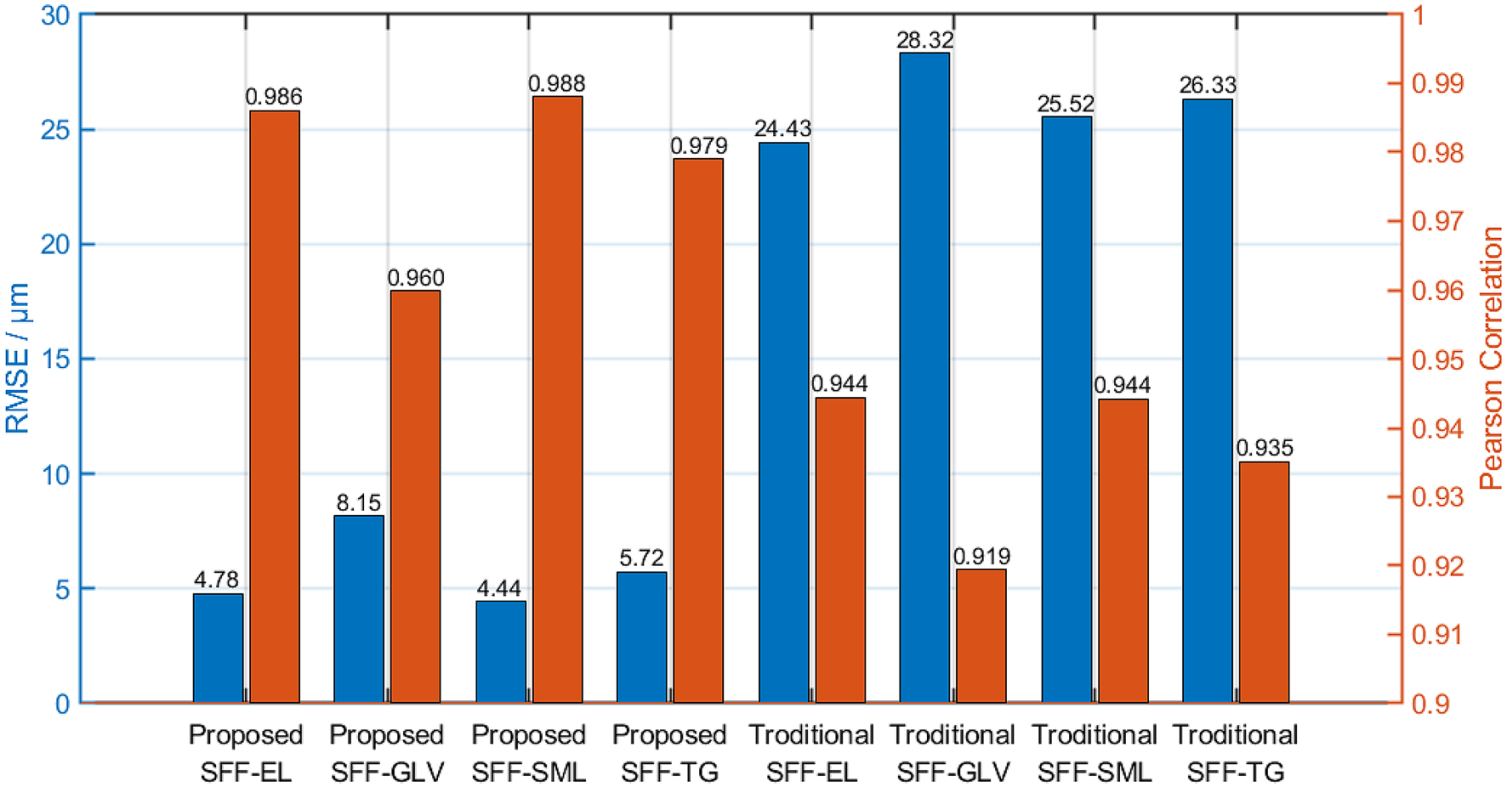
RMSE and Pearson correlation of proposed SFF and traditional SFF using four different focus measure operators

Figure 8 shows the comparison between the proposed SFF method and traditional SFF method concerning RMSE and Pearson correlation with different focus measure operators. It is evident that the proposed SFF method demonstrates superior performance as it features smaller RMSE and more significant correlation. It is believed that the amelioration on distortion contributes to the significant improvement on performance. Moreover, system robustness was improved by the introduction of NaN value and the design of weighted average in focus measure. Regarding the proposed SFF method, the SML operator outperformed the other operators: the RMSE of SML is lower than 4.44 µm. Correlations of the four operators are close to 1, indicating high correlation between the estimated depth and the ground truth, despite the relatively smaller correlation shown by the GLV operator.

Friedman test (a non-parametric hypothesis test) and post-hoc Nemenyi [28] test were adopted as a further evaluation of the focus measure operators. If $r_i$ is the average rank of *i*-th algorithm, and *k* is the number of algorithms, and *N* is number of datasets, the statistic

13 $$\tau _F=\frac{(N-1)\tau _{\chi ^2}}{N\left( k-1\right) -\tau _{\chi ^2}},$$

is subject to *F* distribution, wherein

14 $$\tau _{\chi ^2}=\frac{12N}{k(k+1)}\left( \sum _{i=1}^{k}r_i^2-\frac{k\left( k+1\right) ^2}{4}\right) .$$

Here, the performance of the four operators for each dataset were ranked, and the average rank of each operator was calculated. Table 2 shows rankings of the four operators, and unsurprisingly, the SML operator ranks first in most of datasets. According to Formulas (13, 14), the statistic can be calculated: $\tau _F=26.714$. Taking the significance level $\alpha =0.1$, the critical value of *F* distribution is $t_\alpha =2.490<\tau _F$. Here, null-hypothesis is rejected, and it can be said that performance of the four operators differs significantly.

**Table 2:** Rank table of four operators

Dataset	EL	GLV	SML	TG
$D_1$	2	4	1	3
$D_2$	3	4	1	2
$D_3$	1	4	2	3
$D_4$	1	4	2	3
$D_5$	2	3	1	4
$D_6$	1	4	2	3
$D_7$	3	4	1	2
$D_8$	3	4	1	2
$D_9$	1	4	2	3
$D_{10}$	2	4	1	3
Average rank	1.9	3.9	1.4	2.8

The post-hoc Nemenyi test distinguishes the algorithms by pairs. Two algorithms are significantly different if the difference of corresponding average ranks reaches the critical difference

15 $$\begin{aligned} CD=q_{\alpha }\sqrt{\frac{k\left( k+1\right) }{6N}}. \end{aligned}$$

**Fig. 9:**
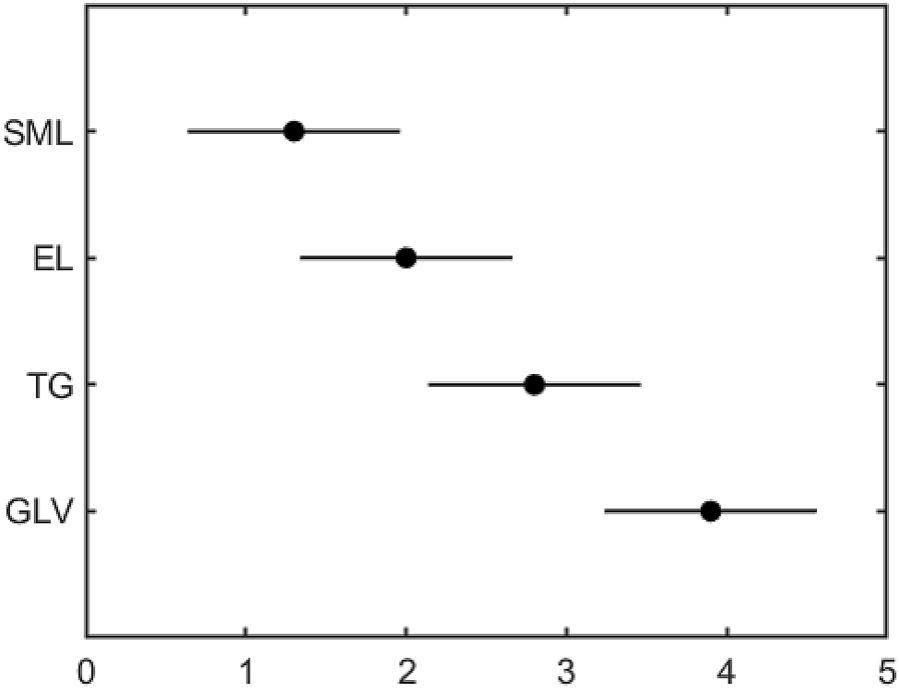
Friedman/Nemenyi test of four focus measure operators in our proposed method

If the confidence is $\alpha =0.1$, the critical value is $q_\alpha =2.291$ according to Tukey distribution. Then the critical difference is $CD=1.323$. Figure 9 shows the Friedman/Nemenyi test of the four focus measure operators. Normally, Laplacian family performs better than the other operators, a fact that agrees with results from previous studies [17]. The SML operator showed the best performance in all datasets among the operators, and its performance differ significantly from that of TG and GLV.

### Discussion on size of local window in focus measure

**Fig. 10:**
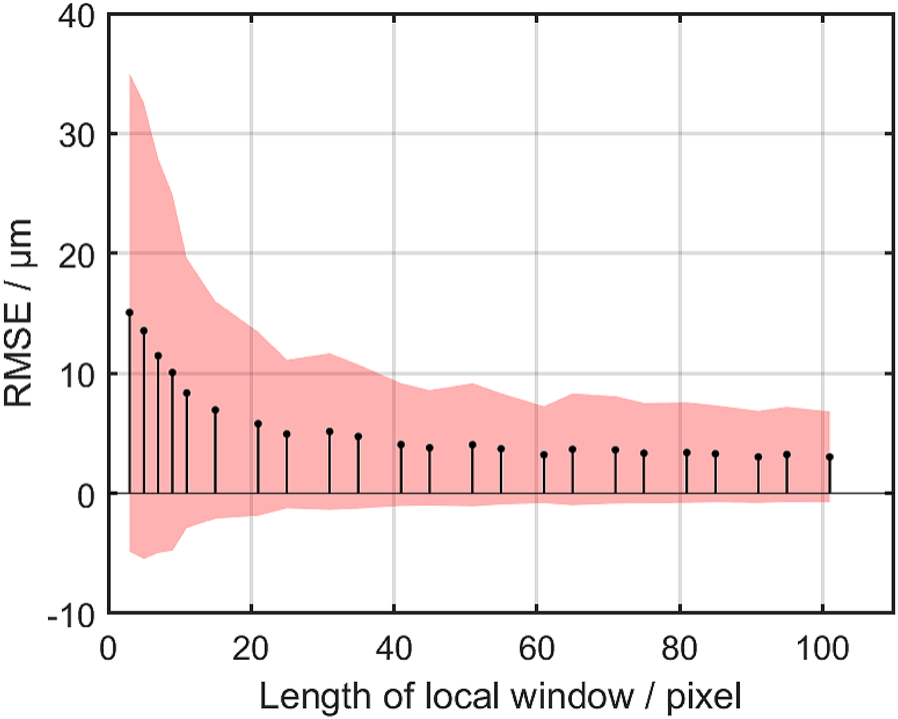
RMSE of height measurements with respect to different length of local window. The red area denotes the standard deviation

The recurrent blur techniques in the algorithm specified in this paper were used as denoising modules, but the lost details may result in low resolution and inaccurate measurements. In subsection *Focus measure*, $\Omega (\cdot )$ is defined as the adjacent region implemented by a local window. The size of local window is a trade-off between denoising and resolution. To find an appropriate window size, RMSE of height measurement with respect to side length of square local window (Fig. 10) was demonstrated. The window side length was tested from 5 to 95 pixels by 10 pixels, and from 11 to 101 pixels by 10 pixels. In addition, size of 3, 7, 9 pixels were included for distinct revealing.

In Fig. 10, a reasonably decreasing trend can be observed concerning RMSE and the corresponding standard deviation as the side length of local window grows. While large window size causes information loss, small window size leads to increased noise, which inhibits the number of valid depth of pixels because of NaN. Therefore, in order to reserve as much information as possible for the sake of high accuracy, it is preferred to select a value where RMSE tends to be flat (e.g. 25 pixels in the algorithm proposed herein).

Though the FM operator SML and dataset $D_5$ are adopted in this subsection as a typical pattern, similar results are also available for other conditions.

## Conclusion

This paper summarized the prevailing methods for measuring leaf surface roughness through a statement of merits and demerits. A new method based on shape from focus was proposed, and the SFF system was established. Different from traditional SFF used in macrocosm, the proposed method requires image registration as preprocessing. According to the experiments, the introduction of distortion correction and focus measure optimization succeeded in increasing the accuracy and robustness. The performance of the 4 focus measure operators were quantified by RMSE and Pearson correlation on the step between the two precise gauge blocks. The proposed method with the SML operator reached the best performance on RMSE and Pearson correlation, which are 4.44 µm and 0.988. Freidman test and post-hoc Nemenyi test also indicated that the SML operator performed best among the 4 operators, exhibiting significant superiority, especially when compared with EL. Roughness measurement of leaf surface based on SFF was not been fully explored, but its potential in plant phenotyping was demonstrated. We believe the method has broad research prospect in the field of plant phenotyping. Future studies may aim to optimize the integration of the SFF system, and to improve the time-consuming algorithm for practical adoption.

## Data Availability

The datasets used or analysed during the current study are available from the corresponding author on reasonable request.
